# Supplementary material for: Analysis of Predicted Amino Acid Sequences of Diatom Microtubule Center Components
Source: Int J Mol Sci. 2023 Aug 14;24(16):12781. doi: 10.3390/ijms241612781 (PMC10454807; doi:10.3390/ijms241612781)
Supplement: Supplementary file 1 [file ijms-24-12781-s001.zip › Supplementary_Table_S1.pdf]

Table S1. Description of GCP sequences identified for diatoms and for out-group.

	Species	Strain	Accession number	GCP	length, a.a.	% identity within aspecies
**Animals**						
	Homo sapiens		NP_001243546	GCP2	930	
			NP_006313	GCP3	907	15,8
			NP_001273343	GCP4	667	10
			AAK77662	GCP5	1024	6,4
			NP_065194	GCP6	1819	5
**Plants**						
	Arabidopsis thaliana		AED92422	GCP2	679	
			Q9FG37	GCP3	838	14,9
			OAP01290	GCP4	745	15,7
**Fungi, Ascomycota**						
	Saccharomyces cerevisiae		QHB09163	Spc97	823	
			AJT23326	SPC98	846	12,4
**Chromista, Heterokonta, Oomycota**						
	Phytophthora nicotianae		KUF64340	GCP2	1210	
			KUF76510	GCP3	931	15,2
			KUF76976	GCP4	850	8,8
			KUG00998	GCP6	1209	8,6
**Chromista, Heterokonta, Ochrophyta**						
	Ectocarpus sp.		CAB1097826	Spc98	938	
**Chromista, Heterokonta, Bacillariophyta, Coscinodiscophyceae**						
	Aulacoseira subarctica	CCAP_1002/5	CAMPEP_0172433114	GCP2	468	
	Corethron pennatum	L29A3	CAMPEP_0200315726	GCP2	491	
	Corethron hystrix	308	CAMPEP_0113304990	GCP2	1025	
			CAMPEP_0113310304	GCP3	415	6,7
	Coscinodiscus wailesii	CCMP2513	CAMPEP_0172503672	GCP3	465	
			CAMPEP_0172505900	GCP4	799	9,7
	Dactyliosolen fragilissimus		CAMPEP_0184873638	GCP3	1022	
			CAMPEP_0184869688	GCP2	811	14
**Chromista, Heterokonta, Bacillariophyta, Mediophyceae**						
	Attheya septentrionalis	CCMP2084	CAMPEP_0198290388	GCP2	750	
	Chaetoceros affinis	CCMP159	CAMPEP_0187021282	GCP2	334	
			CAMPEP_0187012874	GCP3	221	13,1
	Chaetoceros curvisetus		CAMPEP_0187042092	GCP2	557	
			CAMPEP_0187074682	GCP3	316	8
	Chaetoceros debilis	MM31A-1	CAMPEP_0200873290	GCP3	886	
			CAMPEP_0200895134	GCP2	988	13,9
	Chaetoceros dichaeta	CCMP1751	CAMPEP_0198261590	GCP3	572	
			CAMPEP_0198275018	GCP2	198	6,1

Chaetoceros tenuissimus		GFH58980	GCP2	1020	
		GFH48628	GCP3	1006	14,5
Chaetoceros neogracile	CCMP1317	CAMPEP_0200991526	GCP3	1085	
		CAMPEP_0201015168	GCP2	1274	13
Chaetoceros_sp.	GSL56	CAMPEP_0176479560	GCP3	1030	
Cyclotella meneghiniana	CCMP_338	CAMPEP_0172279446	GCP2	1350	
		CAMPEP_0172272694	GCP3	790	8,8
Detonula confervacea	CCMP_353	CAMPEP_0172328552	GCP2	1443	
		CAMPEP_0172325532	GCP3	1003	11,2
Ditylum brightwellii	GSO104	CAMPEP_0193981746	GCP3	423	
		CAMPEP_0193939136	GCP2	523	11,4
	GSO105	CAMPEP_0187353236	GCP4	353	
		CAMPEP_0187334744	GCP3	1090	6,5
		CAMPEP_0187337376	GCP2	804	14,4
Eucampia antarctica	CCMP1452	CAMPEP_0197836786	GCP2	274	
Extubocellulus spinifer	CCMP396	CAMPEP_0200506788	GCP4	733	
		CAMPEP_0200505284	GCP2	1698	8,7
		CAMPEP_0200506102	GCP3	1424	6,3
Leptocylindrus danicus	CCMP1856	CAMPEP_0196824282	GCP3	630	
		AMPEP_0196824264	GCP2	874	14,8
Leptocylindrus danicus var. danicus	B650	CAMPEP_0116004766	GCP6	530	
		CAMPEP_0116007126	GCP3	715	8,1
		CAMPEP_0116024682	GCP2	1111	6,1
Minutocellus polymorphus	H13	CAMPEP_0181031612	GCP2	2129	
		CAMPEP_0181035666	GCP3	1034	8,34
		CAMPEP_0181022260	GCP4	694	3,9
Minutocellus polymorphus	RCC2270	CAMPEP_0185814462	GCP3	780	
Odontella		CAMPEP_0113535984	GCP3	1018	
		CAMPEP_0113560700	GCP4	530	10,8
Proboscia alata	PI-D3	CAMPEP_0200179268	GCP5	359	
		CAMPEP_0200140732	GCP4	695	3,2
		CAMPEP_0200139986	GCP3	861	6,4
		CAMPEP_0200148558	GCP2	1936	2,3
Proboscia inermis	CCAP1064	CAMPEP_0171321380	GCP4	272	
		CAMPEP_0171303342	GCP2	335	13,3
Skeletonema costatum	1716	CAMPEP_0113380788	GCP3	937	
		CAMPEP_0113407062	GCP2	750	14,5
Skeletonema dohrnii	SkelB	CAMPEP_0192137624	GCP3	697	
Skeletonema grethea	CCMP_1804	CAMPEP_0201711052	GCP2	480	
Skeletonema japonicum	CCMP2506	CAMPEP_0201741136	GCP2	797	

			CAMPEP_0201719476	GCP3	966	13,3
	Skeletonema marinoi	SkelA	CAMPEP_0192226214	GCP3	902	
			CAMPEP_0192223726	GCP2	531	9,4
	Skeletonema menzelii	CCMP793	CAMPEP_0192258710	GCP3	946	
			CAMPEP_0192282762	GCP2	844	14,9
	Thalassiosira antarctica	CCMP982	CAMPEP_0200099138	GCP2	792	
			CAMPEP_0200087340	GCP3	985	15,5
	Thalassiosira gravida	GMp14c1	CAMPEP_0200701512	GCP3	399	
			CAMPEP_0200731306	GCP2	881	9
	Thalassiosira miniscula	CCMP1093	CAMPEP_0201090696	GCP2	724	
			CAMPEP_0201084504	GCP3	1188	10
	Thalassiosira oceanica	CCMP1005	AMPEP_0192942200	GCP3	916	
	Thalassiosira pseudonana		262619	GCP2	676	
			6459	GCP3	1092	11,2
	Thalassiosira punctigera	Tpunct2005C2	CAMPEP_0172574286	GCP3	617	
			CAMPEP_0172576886	GCP2	1522	7,6
	Thalassiosira rotula	CCMP3096	CAMPEP_0192961864	GCP2	1551	
			CAMPEP_0192976186	GCP3	1038	10
		GSO102	CAMPEP_0192997528	GCP2	276	
			CAMPEP_0192994958	GCP3	101	8,3
	Thalassiosira weissflogii	CCMP1010	CAMPEP_0193052944	GCP3	1066	
			CAMPEP_0193053784	GCP2	1833	8
		CCMP1336	CAMPEP_0193068758	GCP2	1585	
			CAMPEP_0193084036	GCP3	1066	11,6
**Chromista, Heterokonta, Bacillariophyta, Bacillariophyceae, Urneidophycidae**						
	Asterionellopsis glacialis	CCMP134	CAMPEP_0199907404	GCP2	811	
			CAMPEP_0199883012	GCP3	841	17,8
**Chromista, Heterokonta,Bacillariophyta, Bacillariophyceae, Fragilariophycidae**						
	Ulnaria acus		19284	GCP3	726	
			21600	GCP2	1096	13,3
	Thalassionema frauenfeldii	CCMP_1798	CAMPEP_0178931292	GCP3	548	
	Thalassionema nitzschioides	L26-B	CAMPEP_0200206608	GCP3	758	
	Thalassiothrix antarctica	L6-D1	CAMPEP_0200979464	GCP2	1131	
	Fragilaria crotonensis		KAI2513735	GCP2	1077	
			KAI2505852	GCP3	772	11,1
	Licmophora paradoxa	CCMP2313	AMPEP_0202447606	GCP3	257	
			CAMPEP_0202474432	GCP2	651	7,8
	Staurosira complex sp.	CCMP2646	CAMPEP_0202510880	GCP2	1114	

Chromista, Heterokonta, Bacillariophyceae, Bacillariophycidae

	Amphiprora sp.		CAMPEP_0186515180	GCP2	1019	
	Amphiprora paludosa	CCMP125	CAMPEP_0172461094	GCP2	496	
	Craspedostauros australis	CCMP3328	CAMPEP_0198132742	GCP2	213	
			CAMPEP_0198136308	GCP3	383	10,4
	Cylindrotheca_closterium		CAMPEP_0113642850	GCP3	813	
	Fistulifera solaris		GAX12253	GCP2	1232	
			GAX1175	GCP3	787	11,4
	Fragilariopsis cylindrus		224880	GCP3	918	
			207014	GCP2	756	16,4
	Fragilariopsis kerguelensis	L26 C5	CAMPEP_0188062944	GCP3	944	
			CAMPEP_0188071756	GCP2	1542	9,6
		L2 C3	CAMPEP_0199372024	GCP3	944	
			CAMPEP_0199366584	GCP2	1325	12,6
	Nitzschia inconspicua		KAG7346153	GCP2	1931	
	Nitzschia punctata	CCMP561	CAMPEP_0199321270	GCP3	405	
			CAMPEP_0199329008	GCP2	611	11,1
	Nitzschia sp.		CAMPEP_0113450388	GCP3	679	
			CAMPEP_0113502030	GCP2	509	11
	Pseudo-nitzschia arenysensis	B593	CAMPEP_0116114752	GCP3	935	
			CAMPEP_0116116392	GCP2	1142	15,5
	Pseudo-nitzschia australis	10249	CAMPEP_0199655196	GCP2	717	
			CAMPEP_0199653806	GCP3	993	13,1
	Pseudo-nitzschia delicatissima	B596	CAMPEP_0116098148	GCP3	948	
			CAMPEP_0116101462	GCP2	1489	12,8
	Pseudo-nitzschia fraudulenta	WWA7	CAMPEP_0199816714	GCP2	448	
			CAMPEP_0199759484	GCP3	317	11,3
	Pseudo-nitzschia heimii	UNC1101	CAMPEP_0197197498	GCP2	630	
			CAMPEP_0197199456	GCP3	371	9,3
	Pseudo-nitzschia multiseries		183312	GCP2	973	
			317641	GCP3	876	17,3
	Pseudo-nitzschia pungens		CAMPEP_0172373338	GCP2	230	
	Phaeodactylum tricornutum		44333	GCP3	844	
			47314	GCP2	1275	11,9
